# What has affected the governance effect of the whole population coverage of medical insurance in China in the past decade? Lessons for other countries

**DOI:** 10.3389/fpubh.2023.1079173

**Published:** 2023-03-30

**Authors:** Ting Zhang, Hongyu Zhang, Wenqing Miao, Jinpeng Xu, Qi Shi, Jian Liu, Fangmin Deng, Jingran He, Fangting Chen, Zheng Kang, Qunhong Wu, Guomei Tian

**Affiliations:** ^1^School of Health Management, Harbin Medical University, Harbin, Heilongjiang, China; ^2^Department of Nuclear Medicine, The Fourth Hospital of Harbin Medical University, Harbin, Heilongjiang, China

**Keywords:** medical insurance, population coverage, medical insurance governance, governance effect, influence factors

## Abstract

**Objective:**

This study aimed to explore the current state of governance of full population coverage of health insurance in China and its influencing factors to provide empirical references for countries with similar social backgrounds as China.

**Methods:**

A cross-sectional quantitative study was conducted nationwide between 22 January 2020 and 26 January 2020, with descriptive statistics, analysis of variance, and logistic regression models *via* SPSS 25.0 to analyze the effectiveness and influencing factors of the governance of full population coverage of health insurance in China.

**Results:**

The effectiveness of the governance relating to the total population coverage of health insurance was rated as good by 59% of the survey respondents. According to the statistical results, the governance of the public's ability to participate in insurance (OR = 1.516), the degree of information construction in the medical insurance sector (OR = 2.345), the government's governance capacity (OR = 4.284), and completeness of the government's governance tools (OR = 1.370) were all positively correlated (*p* < 0.05) on the governance effect of the whole population coverage of health insurance.

**Conclusions:**

The governance of Chinese health insurance relating to the total population coverage is effective. To effectively improve the effectiveness of the governance relating to the total population coverage of health insurance, health insurance information construction, governance capacity, and governance tools should be the focus of governance to further improve the accurate expansion of and increase the coverage of health insurance.

## Background

The actualization of health insurance population coverage (HIPC) is one of the biggest challenges facing global health development and is one of the essential health system reforms and development goals advocated by the World Health Organization (WHO) (1). Relevant studies showed that, in 2019, the average effective coverage rate of the global insured population was 60.3% and more than one-third of the world's population still lacked health insurance (2). In the past decade, through its governance of the medical insurance sector, China rapidly increased the average effective coverage rate of its insured population from <30% in 2000 to ~95% in 2010 and has maintained this level to this day (3–6). There is no denying that, as a typical developing country, China has a large population and complex structure where the productivity level is not high, yet, in the shortest possible time, it successfully completed the most expansive HIPC (7, 8). Chinese HIPC governance and its experience, compared with other countries with similar social backgrounds, has received worldwide recognition and high reference value and is worth examining.

Although China has maintained a high level of health insurance population coverage in recent years, still ~80 million people are not covered by health insurance. This phenomenon has continued since 2010. Therefore, to understand the effectiveness of HIPC governance in China in recent past, an in-depth analysis of the factors affecting the efficacy of HIPC governance has become essential for further coverage of the HIPC in China. HIPC governance effectiveness refers to the systematic or singular results produced by the party and government as the core governing body through a series of systematic arrangements and the combined use of composite governance tools and instruments in HIPC. China's efforts in the last decade in the governance of full population coverage of health insurance have led the country to achieve 95% population coverage of health insurance in 2018 alone. However, the special nature of the voluntary principle of participation has impacted the governance of the whole population coverage, calling for the need to focus on the structure of the uninsured population even while acknowledging the increase in the number of people covered by health insurance. Effective governance of health insurance coverage for the entire population should be demonstrated not just by the fact that the majority of the population is insured but also to ensure that the uninsured do not choose to be insured because they are unable to afford health insurance premiums.

Naser Derakhshani (9), Dorjsuren Bayarsaikhan (10), Chhabi L. Ranabhat (11), Gulbiye Yenimahalleli Yasar (12), Ngan Do (13), and other scholars, in a systematic review of the process involved in the HIPC, suggested that social infrastructure, government participation, subject governance capacity, and the choice of instruments and tools were important factors that affect the effectiveness of HIPC governance. A review of the existing studies revealed that most scholars have mainly explored the factors affecting the effectiveness of HIPC governance from the government's perspective. The effectiveness of HIPC governance is also closely related to the governance of individual factors. Studies have shown that the lower affordability of health insurance for the uninsured population is a major factor affecting the achievement of HIPC in most countries such as Vietnam, which has a high uninsured rate of 75% among the poor, and Kenya, which has the highest uninsured rate of 97% among the poor (14–16). The latest data from the China Health and Nutrition Survey (CHNS) also showed that 47% of the uninsured were unable to enroll due to insufficient ability to pay for health insurance, which confirms that it is not only government factors but also individual factors that affect the effectiveness of HIPC governance in China. However, many of the current literature focuses on the influence of specific factors and lacks a comprehensive analysis of HIPC governance from a holistic perspective. Therefore, based on the comprehensive analysis framework of “Government Factors—Governance of Individual Factors,” this study aimed to explore the factors that influence the effectiveness of HIPC governance holistically.

In this study, we aimed to evaluate the governance effectiveness of HIPC in China, comprehensively explore the factors that significantly affect the governance effectiveness of HIPC in China, share important lessons learned from China's move toward HIPC, and provide empirical references for other countries with similar socio-historical backgrounds as China to achieve HIPC in their respective countries in the future.

## Materials and methods

### Study design

This study used a cross-sectional questionnaire to analyze the effectiveness of HIPC governance in China and the factors influencing it. Before the formal survey, a pre-survey was conducted, and 80 questionnaires were collected using a convenience sampling method to improve the quality of our design and questionnaire. The cross-sectional quantitative study was conducted nationwide between 22 January 2020 and 26 January 2020 using a purposive sampling method through the Questionnaire Star platform, a widely recognized online questionnaire platform in China, influenced by the outbreak of coronavirus (COVID-19). For research fit, the sample population eligible for the study had to meet the following conditions: The survey population had to be familiar with health insurance, be fully aware of the dynamics of health insurance policies, and understand the effects of policy implementation. To ensure the representativeness and validity of the original data, managers and staff of health insurance and its related departments, and academics whose research field is health insurance and its related fields were selected as the respondents of the survey, and their questionnaire results were used as the sample data.

The introductory section of the questionnaire required written informed consent before proceeding further. According to the IP address recorded on the questionnaire, each participant could only answer once. If a questionnaire was completed in 8 min or more, which was the minimum time our team tested to complete the questionnaire, and logically answered two questions, it was judged valid and included in the analysis; otherwise, it was removed. In the end, 2,000 questionnaires were distributed in this study and 1,975 valid questionnaires were returned, with an effective rate of 98.75%.

### Data extraction

To measure the governance of HIPC in China, respondents were asked “How effective do you think the governance of universal health coverage is at present?”. The question was evaluated by setting the options to “not at all effective, not very effective, average, relatively effective, very effective.” Using multiple logistic regression, the dependent variable (HIPC governance) was divided into two categories, with 0 indicating not very effective (selecting “not at all effective” and “not very effective”) and 1 indicating effective (selecting “average,” “relatively effective,” “very effective”).

The governance of individual factors included two key areas: governance in the area of participant awareness and governance in the area of participant capacity. In the survey, respondents were asked the following questions to measure the effectiveness of governance in the two areas of participant awareness and participant participation: “What is the level of governance for participant awareness in the HIPC governance process?” (low = 0; high = 1) and “How well is the governance of participation capacity in the HIPC governance process?” (low = 0; high = 1).

The government factor consisted of five major indicators: the degree of clarity of the government's governance functions, the degree of medical insurance information sharing between regions, the degree of information technology construction in the medical insurance sector, the government's governance capacity, and the completeness of governance tools. These factors were assessed through the survey respondents' answers to the following questions, respectively: “Do you think there is clarity in the government's role in the HIPC governance process?” (Unclear = 0; Clarity = 1); “Does your region share health insurance information with other regions?” (not achieved = 0, achieved = 1); “How well do you think China's medical insurance is currently being informalized?”; “How well do you think the government currently governs HIPC in the area of health care?”; and “How complete do you think our HIPC governance tools are?”. The answers to the last three questions ranged from 0 to 5, with higher scores for a higher level of information technology, higher governance capacity, and better completeness of governance tools. In multiple logistic regression, the three dependent variables were scored as 1–2 for low-level, poor capacity, and incomplete groups, 3 for medium-level, general capacity, and general completeness groups, and 4–5 for high-level, high capacity, and completeness groups.

### Variable selection

#### Control variable

HIPC governance effectiveness, as a subjective evaluation, is influenced by personal characteristics such as personal preference and temperament. Considering the representativeness of the sample, this study selected individual characteristic variables, controlling for gender, age, education, profession, and type of unit. The specific variable assignments are shown in Table 1.

**Table 1:** Selection and description of variables.

**Variable**	**Control group**
**Dependent variable**	
HIPC governance status	0 = bad; 1 = good
**Control variable**	
Sex	0 = Men; 1 = Women
Age	Numerical variable
Educational level	1 = Tertiary and below; 2 = Undergraduate degree; 3 = Masters Degree; 4 = Doctoral degree
Type of profession	0 = Non-Medicare related professions; 1 = Medical insurance or medical insurance-related professions
Type of unit	0 = Medical insurance sector; 1 = Medical insurance related departments
**Explaining variable**	
**Governance of individual factors**	
Governance of the population's awareness of insurance participation	0 = low; 1 = High
Governance of people's ability to participate in insurance	0 = low; 1 = High
**Government factors**	
Clarity of government's governance functions	0 = Unclear; 1 = Clarity
The degree of medical insurance information sharing between regions	0 = Not achieved; 1 = Achieved
The degree of information technology construction in the medical insurance sector	1 = Low level; 2 = General; 3 = High level
Government's governance capacity	1 = Poor ability; 2 = General; 3 = High ability
Governance tools completeness	1 = Incomplete; 2 = General; 3 = Complete

#### Explaining variable

In this study, HIPC governance was used as the dependent variable. The explanatory variables mainly included (1) governance of individual factors, including governance of the population's awareness of insurance participation and governance of the population's ability to participate in insurance; and (2) government factors, which included the degree of clarity of the government's governance function, the degree of medical insurance information sharing between regions, the degree of information technology construction in the medical insurance sector, the government's governance capacity, and the completeness of governance tools. The specific distribution of variables is shown in Table 1.

### Statistical analysis

#### Logistic regression model

Logistic regression is a generalized linear regression analysis that uses a logit function to fit data to predict the probability of an event occurring or to screen for joint markers or dependent variables, with the probability and independent variable model usually being an S-shaped curve. Since the dependent variables used in this study were binary (0 and 1), the binary logistic regression model, which is mainly used to describe an optimal mapping relationship between the independent variables and the response variables with dichotomous nature, was used. The expression of the logistic regression mathematical model was as follows (17–19):


$$\begin{array}{l} \;logit\{y_{i}=y_{1}|X_{i}\}=logit(p_{i})=\log\left[\frac{p_{i}}{1-p_{i}}\right]\\ =\beta_{0}+\beta_{1}X_{1}+\cdots +\beta_{j}X_{j}+\cdots +\beta_{k}X_{k}=\beta_{0}+\beta^{{}^{\prime }}X_{i}, \end{array}$$

where *p*_*i*_ = *probability* {*y*_*i*_ = *y*_1_|*X*_*i*_}, β_0_ is the *y* intercept, β′ is the vector of slope parameters, *y*_*i*_ is the first ordered level of *y*, and *X*_*i*_ is the vector of explanatory variables.

In the binary logistic regression equation above, the response variable was the log dominance ratio (log) for Y = 1. The meaning of the regression coefficient can be understood as the change in the log dominance ratio of the dependent variable that may be induced by a unit change in the independent variable, i.e., the parameter in the regression equation that indicates the magnitude of the effect of the independent variable on the dependent variable. As the logarithmic function is more convenient, faster, and easier to interpret, it was chosen as the model for statistical analysis in this study to determine the factors affecting the effectiveness of HIPC governance.

#### Data analysis

All statistical analyses were performed using SPSS 26.0, with confidence intervals at 95%, and bilateral statistical significance was based on a *p*-value of <0.05. In the first step, descriptive statistical analysis was used to describe participant characteristics and HIPC governance effects; in the second step, chi-square independence tests were used to analyze differences in categorical variables; and in the third step, multi-factor logistic regression analysis was performed to identify significant factors influencing HIPC governance effects and to control for possible confounding variables.

## Results

### The characteristics of the sample

Among the control variables, men accounted for 32.05%, and women for 67.95% of respondents; the respondents were all adults, with a majority aged 44 years and below (66.73%). The lowest level of education among the group was bachelor's degree (56.10%), indicating that the education level of the respondents was in line with the actual situation of the nine-year compulsory education implemented in China. Most of the survey respondents' majors were in medical insurance-related disciplines, accounting for 53.6% of respondents; their job types were mainly in the medical insurance sector, accounting for 81.1% of respondents, indicating that most of the survey respondents' job types were directly related to medical insurance.

### Single-factor analysis of the governance effect of full population coverage of medical insurance

**Figure 1:**
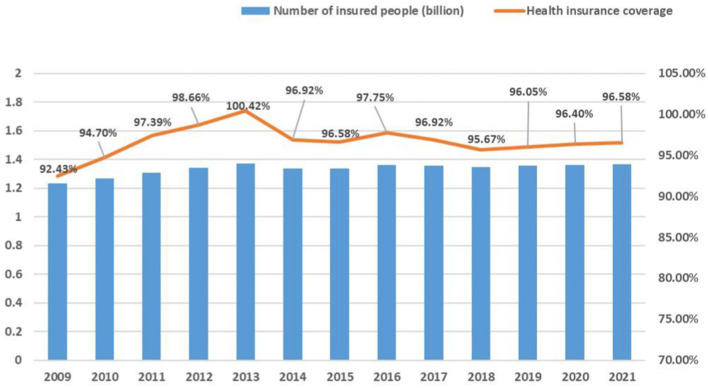
Trend of health insurance coverage in China from 2009 to the present. In 2013, due to the duplication of statistics on the number of participants in the New Rural Cooperative and urban medical insurance, the total number of people covered by the national medical insurance was high, and if duplicate statistics were excluded, the participation rate would still be above 95%.

The results of the survey showed that 59% of the respondents had a good knowledge of the governance efficiency of the HIPC. The governance effect of HIPC and its influencing factors are detailed in Table 1. The HIPC had achieved remarkable results since the government began governing health insurance coverage for the entire population. Refer to Figure 1 for details.

Table 2 illustrates the estimation results controlling for individual background characteristics with government factors and governance of individual factors as the main explanatory variables. The results showed that all individual factors have a significant effect (*p* < 0.05) on the effectiveness of HIPC governance, and all the governing factors are significantly associated with the efficacy of HIPC governance (*p* < 0.05), except for inter-regional health insurance information sharing. For details, refer to Table 2.

**Table 2:** Sample characteristics and univariate analysis of the effect of governance on health insurance coverage for the entire population.

**Variables**	**Total (*N* = 1,975)**	**Achieving the governance effect of universal health insurance coverage [*****n*** **(%)]**		**X^2^**	***P*-value**
		**Bad**	**Good**		
**Governance of the population's awareness of insurance participation**				13.950	0.000
Unrelated	1,047	385 (0.368)	662 (0.632)		
Related	928	418 (0.45)	510 (0.55)		
**Governance of people's ability to participate in insurance**				12.655	0.000
Unrelated	999	445 (0.445)	554 (0.555)		
Related	976	358 (0.367)	618 (0.633)		
**Clarity of government's governance functions**				8.460	0.004
Unclear	712	259 (0.364)	453 (0.636)		
Clarity	1,263	544 (0.431)	719 (0.569)		
**The degree of medical insurance information sharing between regions**				0.048	0.827
Not achieved	504	207 (0.411)	297 (0.589)		
Achieved	1,471	596 (0.405)	875 (0.595)		
**The degree of information technology construction in the medical insurance sector**				526.250	0.000
Low level	294	184 (0.626)	110 (0.374)		
General	730	483 (0.662)	247 (0.338)		
High level	951	136 (0.143)	815 (0.857)		
**Government's governance capacity**				606.249	0.000
Poor ability	146	105 (0.719)	41 (0.281)		
General	755	529 (0.701)	226 (0.299)		
High ability	1,074	169 (0.157)	905 (0.843)		
**Governance tools completeness**				221.768	0.000
Incomplete	666	323 (0.485)	343 (0.515)		
General	692	378 (0.546)	314 (0.454)		
Complete	617	102 (0.165)	515 (0.835)		

### Multivariate logistic regression analysis of the governance effects of full population coverage of medical insurance

Based on the results of the single-factor analysis, this study assigned the statistically significant influencing factors and the effect of governance of China's health insurance coverage for the whole population in turn. The study substituted them into the logistic regression equation for multi-factor analysis; the results are shown in Table 3 in detail. The effect of targeting the governance of the population's ability to participate and the governance of the health insurance coverage across the population is positively affected at a significant level of 5%. Other indicators positively affect the effect of governance of China's health insurance total population coverage, among which the degree of information construction of the health insurance sector, government's governance ability, and governance tools positively affect at a 5% significant level, that is, the higher the degree of information construction of health insurance sector, the higher the government's governance ability; and the more complete the governance tools and instruments, the better the effect of governance of China's health insurance total population coverage.

**Table 3:** The logistic regression analysis of factors influencing the effectiveness of governance for full population coverage of medical insurance (*n* = 1,975).

**Variable**	**OR (95% CI)**	***P*-value**
Governance of the population's awareness of insurance participation	0.847 (0.677, 1.059)	0.145
Governance of people's ability to participate in insurance	1.516 (1.212, 1.897)	0.000
Clarity of government's governance functions	0.933 (0.738, 1.181)	0.565
The degree of information technology construction in the medical insurance sector	2.345 (1.973, 2.788)	0.000
Government's governance capacity	4.284 (3.490, 5.258)	0.000
Governance tools completeness	1.370 (1.182, 1.588)	0.000

### Sensitivity analysis

This study conducted a sensitivity analysis by changing the outcome classification of the independent or dependent variables, which may have an impact on the significant results of the explanatory variables. Therefore, in this study, the results of the independent variables were first set to 3 categories, i.e., 1 = not very effective (choice of “not at all effective” and “not very effective”), 2 = average (choice of “average”) and 3 = effective (choice of “relatively effective” and “very effective”), the results of the explanatory variables were statistically analyzed according to the 3 categories, and the results of the adjusted model were unchanged (Table 4). Subsequently, the independent variables were analyzed by the two classification results, the explanatory variables were analyzed by the five classification results of the original data, and the statistical steps were repeated. The adjusted model results were generally consistent with the statistical analysis of the previous data, again indicating that the results of this study were stable and reliable (Table 5).

**Table 4:** Classification results according to the explaining variable of the original data—logistic regression analysis (*n* = 1,975).

**Variable**	**OR (95% CI)**	***P*-value**
Awareness of people's participation	0.835 (0.669, 1.044)	0.113
Crowd participation capacity	1.505 (1.204, 1.882)	0.000
Clarity of government's governance functions	0.940 (0.743, 1.189)	0.607
The degree of information technology construction in the medical insurance sector	2.088 (1.792, 2.432)	0.000
Government's governance capacity	3.598 (2.996, 4.320)	0.000
Governance tools completeness	1.288 (1.125, 1.474)	0.000

**Table 5:** Analysis of results classified according to explaining variable 3—logistic regression analysis (*n* = 1,975).

**Variable**	**Inefficient**		**General**	

	**OR (95% CI)**	***P***	**OR (95% CI)**	***P***
**Governance of the population's awareness of insurance participation**				
Unrelated	0.815 (0.508, 1.307)	0.396	0.841 (0.659, 1.074)	0.166
Related (reference)				
**Governance of people's ability to participate in insurance**				
Unrelated	1.212 (0.752, 1.955)	0.430	1.598 (1.248, 2.045)	0.000
Related (reference)				
**Clarity of government's governance functions**				
Unclear	0.581 (0.345, 0.981)	0.042	0.971 (0.753, 1.253)	0.823
Clarity (reference)				
**The degree of medical insurance information sharing between regions**				
Not achieved	2.482 (1.490, 4.133)	0.000	1.101 (0.828, 1.463)	0.510
Achieved (reference)				
**The degree of information technology construction in the medical insurance sector**				
Low level	12.558 (6.416, 4.579)	0.000	2.526 (1.731, 3.685)	0.000
General	3.086 (1.523, 6.254)	0.002	4.972 (3.734, 6.621)	0.000
High level (reference)				
**Government's governance capacity**				
Poor ability	11.054 (5.630, 1.704)	0.000	4.076 (2.478, 6.702)	0.000
General	2.452 (1.336, 4.499)	0.004	5.448 (4.167, 7.124)	0.000
High ability (reference)				
**Governance tools completeness**				
Incomplete	2.014 (1.027, 3.950)	0.042	2.139 (1.526, 2.999)	0.000
General	1.477 (0.710, 3.073)	0.297	2.567 (1.840, 3.582)	0.000
Complete (reference)				

## Discussion

This study aimed to investigate the factors that influence the effectiveness of the governance of health insurance total population coverage. The results of the study showed that factors such as the governance of the population's ability to participate in insurance, the degree of information technology construction in the medical insurance sector, government's governance capacity, and governance tools completeness significantly affect the effectiveness of the governance of health insurance coverage for the whole population.

This study found that governance of the population's ability to participate in insurance was positively correlated with the impact of achieving governance effectiveness in HIPC. Studies have shown that nearly half of China's uninsured population is uninsured due to their low participation ability. This group offers a high willingness to participate, resulting in an unbalanced phenomenon of high demand for health insurance and adverse selection of actual participation (20–22). Studies have shown that, in other countries, the uninsured are more likely to be uninsured due to their low capacity to participate (23, 24). China noticed this phenomenon early in the process of HIPC promotion and has enhanced the public's ability to participate in health insurance, especially for the poor, through diversified means such as health insurance poverty alleviation policies, medical assistance underwriting policies, and continuous lowering of health insurance contribution thresholds, effectively bringing people with low participation ability into the scope of health insurance, making China's HIPC governance achieve good results (21, 25). According to official data, since China launched medical insurance to help the poor and established a multi-level medical insurance system, 230 million people in poverty have been insured, and the coverage of the poor has stabilized at over 99.9% (26). Therefore, China should adhere to the policy guidelines such as “health insurance for the poor” and continue the evolution of the governance measures for the population's ability to participate in the insurance and continue to consolidate the achievements of “universal health insurance, Everyone who can be guaranteed is guaranteed” in China.

Second, the degree of information technology construction in the medical insurance sector significantly impacts the effectiveness of achieving full population coverage of medical insurance governance. Tomasz Janowski's research also shows that the development of information technology and digitalization affects governance goals (27). Countries such as Brazil have effectively promoted HIPC by adopting a unified medical insurance information system platform (28). Currently, China's medical insurance information technology is in its infancy. The medical insurance information system cannot share information with other related departments, making it difficult to effectively and timely screen uninsured people and bring them into the medical insurance coverage from a technical level (29–31). Meanwhile, medical insurance information systems are usually oriented toward regional health insurance services (31), and there are barriers to information systems between different medical insurance systems. This results in the inability to share information on public participation, the increased probability of biased targeting of uninsured people by health insurance departments, and the increased difficulty of full population coverage of medical insurance, which in turn affects the effectiveness of the governance of full population coverage of medical insurance in the country. Therefore, China should hasten the construction of a unified national health insurance information platform, break the barriers between regional health insurance information systems, actively promote the integration and docking of health insurance information systems with information systems of other relevant departments, rely on blockchain and other technologies to achieve inter-regional and inter-departmental sharing of insurance information, establish an identification system for uninsured people, and achieve accurate targeting of uninsured people through big data matching and other means.

Our findings showed that the size of the government's governance capacity and the completeness of its governance tools directly correlates with the effectiveness of the governance of the whole population coverage of health insurance in China. Studies by related scholars also emphasize that the government's governance capacity and the completeness of governance tools are paramount to enhancing the effectiveness of HIPC governance (32–34). Relevant studies have shown that governance in the public sphere, central government institutions, and governance capacity are critical prerequisites for effective governance (35). China has also gradually recognized this in HIPC governance and established the National Health Insurance Administration in 2018 to strengthen the top-level design of HIPC and rationalize the health insurance management system. The former Health and Planning Commission was responsible for the new agricultural cooperation. The former Ministry of Human Resources and Social Security was responsible for the medical insurance of urban residents and urban workers; three kinds of medical security were unified under the management of the Medical Insurance Bureau. From an institutional perspective, it solved the long-standing state of HIPC governance of nine dragons, effectively circumvented the dead ends in HIPC governance, and provided a solid and robust organizational guarantee for HIPC governance in China. In addition, China also provided a good platform and legal support for HIPC governance in China by building a medical insurance information and business coding platform, continuously improving its medical insurance legal system and other diversified governance tools. Its efforts in augmenting its governance capacity and governance tools have effectively alleviated China's HIPC governance dilemma and laid a solid foundation for it to achieve more effective HIPC governance. In recent years, the new pneumonia epidemic has accelerated the process of building digital health insurance in China. In addition, the integration of modern information technology such as blockchain, the Internet of Things, and big data with the health insurance governance system has become the mainstream trend to enhance governance capacity and improve governance tools in China. However, it is still in its initial stage of development and needs to be explored to enhance digital governance capacity and improve digital governance tools. Therefore, China should continue to strengthen its macro system construction and planning, plan for the development of governance capacity and tools, respond positively to the needs of HIPC governance, build a digital governance network for health insurance, strengthen modern information technology, and provide strong institutional and technical support for the development of health insurance governance capacity and tools.

## Conclusions

In this study's survey, 59% of respondents believe that HIPC governance is effective. China has effectively solved the problem of low-participation ability and people's participation through medical insurance poverty alleviation, medical assistance underwriting policy, and lowering the threshold of medical insurance payment. It has also actualized the precise expansion of medical insurance coverage for the uninsured, resulting in its HIPC governance achieving good results. In addition, in realizing HIPC governance, accelerating the construction of health insurance information systems, promoting the modernization of governance capacity, and expanding good governance tools that can effectively promote the accurate expansion of health insurance population coverage, which is an effective path to realizing efficient HIPC governance.

## Limitations

Although this study contributes to the understanding of HIPC governance in China and the factors influencing it, there are still some limitations that need to be noted. China's HIPC governance is the focus of China's current healthcare development, and it has gradually entered a period of rapid development with the continuous improvement of the healthcare governance system, and because the data used in this study is a cross-sectional study for the whole country, it cannot show a longitudinal analysis of the effectiveness of China's health care governance; therefore, the findings of this study may show certain stage characteristics.

## Data availability statement

The data analyzed in this study is subject to the following licenses/restrictions: The datasets generated during and/or analyzed during the current study are not publicly available due to protection of privacy of respondents, but are available from the corresponding author on reasonable request. Requests to access these datasets should be directed to kangzheng@hrbmu.edu.cn.

## Ethics statement

The studies involving human participants were reviewed and approved by Ethics Committee of Harbin Medical University. The patients/participants provided their written informed consent to participate in this study.

## Author contributions

TZ and WM conducted and planned the study and designed the methodology. TZ and HZ drafted the manuscript. TZ and JX conducted a feasibility analysis and helped conceptualize the project. QS and JL conducted the literature search and data visualization. FD and JH proofread and revised the article's language. FC and ZK revised the manuscript and gave critical feedback. ZK and GT conducted quality control and review of the manuscripts. TZ, WM, HZ, and JX contributed to the concept, design, research, data analysis, and drafting of the article. All authors have read and approved the manuscript for publication.
